# Profile of collagen prolines level of anterior rectus sheath tissue in indirect inguinal hernia: A cross-sectional study

**DOI:** 10.1016/j.amsu.2021.102546

**Published:** 2021-07-16

**Authors:** Suluh darmadi, Warsinggih, Mappincara, Joko Hendarto, Ibrahim Labeda, Ronald Erasio Lusikooy, Samuel Sampetoding, Muhammad Iwan Dani, Muhammad Ihwan Kusuma, Julianus Aboyaman Uwuratuw, Erwin Syarifuddin, Muhammad Faruk

**Affiliations:** a Division of Digestive, Department of Surgery, Faculty of Medicine, Hasanuddin University, Makassar, Indonesia; b Department of Public Health and Preventive Medicine, Faculty of Medicine, Hasanuddin University, Makassar, Indonesia; c Department of Surgery, Faculty of Medicine, Hasanuddin University, Makassar, Indonesia

**Keywords:** Inguinal hernia, Proline, Collagen, Age, Body mass index, Cross-sectional study

## Abstract

**Introduction:**

A hernia is a protrusion of an organ or tissue through an abnormal anatomical channel or opening. Epidemiological data indicated an increased prevalence of inguinal hernias in patients with connective tissue diseases. The biomechanical strength of connective tissue is highly dependent on the constituent of the matrix, including collagen. Fibroblasts produce and secrete procollagen containing high concentrations of proline and lysine. Collagen integrity plays an essential role in preventing hernia formation in the abdominal wall. To investigate the relationship between collagen proline levels of the anterior rectus sheath tissue in patients with lateral inguinal hernias (indirect inguinal hernia).

**Methods:**

The study participants consisted of 67 inguinal hernia patients. A sample of anterior rectus tissue was obtained at the time of surgery, then being washed in a PBS buffer (pH 7.4). The measurement of collagen proline levels was subsequently carried out with enzyme linked immunosorbent assay (ELISA).

**Results:**

All study participants were male with mean age of 44 years, mean body mass index of 22.6 kg/m^2^ and mean onset of events of 27 months. Study subjects with reducible, irreducible, and incarcerated hernias were 45.7% (44/67 cases), 14.9% (10/67) and 19.4% (13/67), respectively. The mean proline level of the study subjects was 9.20. Correlation tests showed a correlation of proline levels and age (p = 0.001), body mass index (p = 0.006), and the onset of events (p = 0.023). Meanwhile, correlation of proline levels and occupation (p = 0.235) and clinical degree (p = 0.164) were not statistically significant.

**Conclusion:**

Presence if relationship between proline levels with age, and onset of incidence among indirect inguinal hernia patients.

## HIGHLIGHT

• Collagen is the main structural protein in the muscle wall layer.
• Defects in collagenases will result in abnormal collagen synthesis leading to pathological collagen degradation.
• Decreases in hydroxyproline and collagen were observed in the fascia and muscle tissue of patients with inguinal hernias.
• There are a relationship between proline levels with age, and onset of incidence among indirect inguinal hernia.

## 1 Introduction

Collagen is the main structural protein in the muscle wall layer, accounting for 30% of the dry tissue weight [1]. Defects in collagenases will result in abnormal collagen synthesis or increased protease activity leading to pathological collagen degradation [2]. As a result, there will be a decrease in the tensile strength of the tissue [3]. A condition called lathyrism, presence of abnormal cross-linking of collagen, is associated with herniation. Decreases in hydroxyproline and collagen were observed in the fascia and muscle tissue of patients with inguinal hernias [4,5]. The fibroblasts isolated from these patients also exhibited a proliferative defect and decreased ability to translocate hydroxyproline. In addition, abnormalities in the ratio of various types of collagen, decreased cross-linking and increased solubility of collagen were also observed [6]. A study showed a twofold increase in immature type III collagen in the abdominal wall tissue of patients with inguinal hernias, when compared with non-herniated patients [7,8].

Previous studies evaluating connective tissues in inguinal hernia patients with an electron microscopy of the anterior rectus sheath observed thinner collagen fibrils than normal controls [9,10].

Given that no such studies had been conducted in Indonesia, this study aimed to investigate the profile of collagen proline levels in anterior rectus sheath tissue in patients with indirect inguinal hernia. The results if this study is expected to enhance understanding about the importance of collagen in preventing the incidence of hernias, predicting the possibility of inguinal hernia recurrence in hernia patients who undergo surgery.

## 2 Methods

This cross-sectional study was conducted at Wahidin Sudirohusodo Hospital in Makassar, Indonesia, following a protocol approved by our institutional ethics committee (registration number: 569/UN4.6.4.5.31/PP36/2020) and has been registered with the Research Registry (no. 6808). Herein, we report our work in accordance with the criteria of Strengthening the Reporting of Cohort Studies in Surgery [11].

### 2.1 Population and sample

This study is an observational study with cross-sectional approach. The study population were patients with indirect inguinal hernias admitted to the hospital for herniorrhaphy surgery.

### 2.2 Tissue samples

The muscle tissue of the anterior rectus sheath was removed during surgery. The tissue was then being washed, chopped and homogenized in a PBS solution (pH 7.4) with the vortex. The finished solution was subsequently stored at −20 °C. centrifugation at a speed of 2000–3000 RPM for 15–20 min was conducted afterwards. Finally, the proline collagen level was checked using the enzyme linked immunosorbent assay (ELISA) kit (catalog no. E2431Hu) reagents from the Bioassay Technology Laboratory (Shanghai, China) according to the manufacturer's instructions [[12], [13], [14]].

### 2.3 Statistical analysis

The collected data were analyzed using the Statistical Package for the Social Sciences for Windows (version 23.0; IBM Corp, Armonk, NY). The normality test was carried out on the proline levels using the Kolmogorov-Smirnov test because the data were more than 50. Bivariate analysis in this study aimed to examine the correlation between proline levels and the incidence of lateral inguinal hernia. Correlation test was performed using the Spearman's correlation test if the data distribution was not normal. Statistically significant P values were less than 0.05.

## 3 Results

### 3.1 Study subject characteristics

All the study subjects were male (100%), with the youngest age of 19 years old and the oldest age of 70 years old. The mean body mass index (BMI) of the study subjects were 22.6 kg/m^2^. The mean proline level of hernia tissues examined was 9.20, with the lowest and the highest level of 3.68 and 12.65, respectively (Table 1).

**Table 1:** Baseline characteristics of study subjects.

Variables	Min.	Max.	Mean	SD
Age (years)	19.00	70.00	44.3881	10.76323
Onset (months)	3.00	90.00	27.6866	18.46636
BMI/body mass index (kg/m^2^)	18.70	27.60	22.5940	1.68980
Proline (pg/mL)	3.68	12.65	9.2024	1.89039

### 3.2 Age and proline levels

In this study, the Spearman's test was carried out and the value of p = 0.001 (p < 0.05) was obtained, implying a statistically significant relationship between age and proline levels with a correlation coefficient of −38.6% (moderate significance strength), indicating an inverse relationship. The higher the age, the lower the proline level (Table 2).

**Table 2:** The relationship between age, BMI, clinical onset and proline levels in indirect inguinal hernia patients.

Variables	r	n	p
Age	−0.386	67	0.001
BMI	0.23125	67	0.006
Onset	−0.278	67	0.006
Clinical grade	0.119444	67	0.113889

### 3.3 Body mass index and proline levels

The relationship between body mass index and proline levels resulted in a p value of 0.006 (p < 0.05), indicating a statistically significant relationship between body mass index and proline levels with a correlation coefficient of 33.3% (moderate significance strength), implying a significant correlation between body mass index and proline levels. The relationship is linear, the higher the body mass index, the higher the proline level (Table 2).

### 3.4 Onset of hernia and proline levels

The relationship between onset of the incidence of hernia with proline levels showed a p-value of 0.023 (p < 0.05), indicating a statistically significant relationship between the onset of hernia and proline levels with a correlation coefficient of −27.8% (weak meaningful strength). The variables were inversely correlated, the longer the onset of the hernia incidence, the lower the proline level (Table 2).

### 3.5 Occupations and proline levels

To investigate the relationship between occupations and proline levels, the F ANOVA test was performed, yielding a p value of 0.235 (p > 0.05), indicating no statistically significant relationship. However, mean proline levels appeared to be the lowest among labor group (Table 3).

**Table 3:** The relationship between occupations and proline levels in indirect inguinal hernia patients.

Occupations	N	Mean	SD	Min.	Max	p*
Labor	7	8.38	1.19	6.63	9.61	0.235
Students	19	8.77	2.27	3.68	12.49	
Civil servants	27	9.38	1.97	4.52	12.65	
Business men	14	9.86	1.19	7.70	11.99	
Total	67	9.20	1.89	3.68	12.65	

* Annova test.

### 3.6 Clinical grade and proline levels

To investigate the relationship between clinical grade and proline levels, Spearman's test was performed, resulted in a p value of 0.164 (p > 0.05), indicating no statistically significant relationship.

## 4 Discussion

Hernia is a multifactorial disease involving endogenous and exogenous factors [15]. Changes in collagen composition contributes to hernia development, associated with increased collagen breakdown. Collagen composition appears to affect changes in fascial and systemic tissue. Collagen alterations were also found in patients with recurrent hernias [16,17].

Proline is a non-essential amino acid that is synthesized from glutamate in a two-step pathway that requires nicotinamide adenine dinucleotide phosphate [18,19]. An experimental study on aging mice observed decreased proline, alanine, serine, tyrosine and methionine in plasma with aging [20]. Taniguchi et al. (2006) also reported that changes in the structure and levels of collagen are strongly affected by age [9,21,22].

A previous study conducted by Guevara et al. evaluated the amino acid profile of overweight and obese subjects. The study assigned the subjects into three groups according to their BMI, namely normal weight (20 to <25 kg/m^2^), overweight (25 to < 30 kg/m^2^) and obesity (30 kg)/m^2^). The study found lower levels of glycine and serine, while alanine, aspartate, cysteine, ornithine, phenylalanine, proline and tyrosine levels were found to be higher in overweight or obese young adults compared to normal BMI young adults [23,24].

A study conducted by Taniguchi et al., in 2006 showed that changes in tissue structure in adult hernia patients were associated to secondary pathogenesis of collagen metabolism which ultimately affected both collagen levels and proline levels in the supporting tissue in the inguinal sheath. The primary pathogenesis was related to the incidence of vaginal process pathogenesis triggering indirect inguinal hernias [22,25,26].

Ljungdahl et al. reported no significant relationship between the occupations and development of hernia [27]. Samir et al. argued emphatically that nothing in the medical literature supports the notion that patients with inguinal hernias are unable to lift heavy objects [27]. Wantz et al. also stated that strenuous physical activity alone would not lead to primary or recurrent inguinal herniation [22,28,29].

In a patient with a direct inguinal hernia, Wagh et al. found that the anterior rectus sheath over the defect was thinner than usual, which was due to a decrease in collagen especially hydroxyproline levels (by 19.2%). This may be due to decreased fibroblast proliferation, as demonstrated by fibroblast culture taken from the anterior rectus sheath [30].

The limitations of the study were the small sample sizes, limited data collection period, and the BMI values of the patients were normal or around normal, so there was no significant variability to make valuable conclusion in the relationship between proline level and BMI in indirect inguinal hernia patients.

## 5 Conclusion

This study showed a significant relationship between age, and the onset of indirect inguinal hernias with proline levels. However, occupation and clinical degree showed no relationship with proline levels in indirect inguinal hernia patients.

## Ethical approval

All procedure for human experiment has been approved by Ethics Commission Faculty of Medicine, Hasanuddin University number: 569/UN4.6.4.5.31/PP36/2020.

## Sources of funding

None.

## Author contribution

All authors made a significant contribution to the work reported, whether that is in the conception, study design, execution, acquisition of data, analysis and interpretation, or in all these areas; took part in drafting, revising or critically reviewing the article; gave final approval of the version to be published; have agreed on the journal to which the article has been submitted; and agree to be accountable for all aspects of the work.

## Registration of research studies

This research has been registered with the Research Registry number: 6808.

https://www.researchregistry.com/register-now#home/registrationdetails/609b7598e0659b001be5faf3/

## Guarantor

Warsinggih.

## Consent

The research was conducted ethically in accordance with the World Medical Association Declaration of Helsinki. The patients have given their written informed consent on admission to use their prospective data base and files for research work.

## Provenance and peer review

Not commissioned, externally peer-reviewed.

## Declaration of competing interest

The authors declare that they have no conflict of interests.
